# Patient Portals to Support Palliative and End-of-Life Care: Scoping Review

**DOI:** 10.2196/28797

**Published:** 2021-09-16

**Authors:** M Pilar Ingle, Cristina Valdovinos, Kelsey L Ford, Shou Zhou, Sheana Bull, Starlynne Gornail, Xuhong Zhang, Susan Moore, Jennifer Portz

**Affiliations:** 1 Graduate School of Social Work University of Denver Denver, CO United States; 2 David Geffen School of Medicine University of California Los Angeles Los Angeles, CO United States; 3 Colorado School of Public Health University of Colorado Aurora, CO United States; 4 Department of General Internal Medicine University of Colorado Aurora, CO United States

**Keywords:** patient portal, electronic health record, digital health, palliative care, end-of-life care

## Abstract

**Background:**

Although patient portals are widely used for health promotion, little is known about the use of palliative care and end-of-life (PCEOL) portal tools available for patients and caregivers.

**Objective:**

This study aims to identify and assess the user perspectives of PCEOL portal tools available to patients and caregivers described and evaluated in the literature.

**Methods:**

We performed a scoping review of the academic literature directed by the PRISMA (Preferred Reporting Items for Systematic Reviews and Meta-analyses) extension for Scoping Review and searched three databases. Sources were included if they reported the development or testing of a feature, resource, tool, or intervention; focused on at least one PCEOL domain defined by the National Coalition for Hospice and Palliative Care; targeted adults with serious illness or caregivers; and were offered via a patient portal tethered to an electronic medical record. We independently screened the titles and abstracts (n=796) for eligibility. Full-text (84/796, 10.6%) sources were reviewed. We abstracted descriptions of the portal tool name, content, targeted population, and reported user acceptability for each tool from included sources (n=19).

**Results:**

In total, 19 articles describing 12 tools were included, addressing the following PCEOL domains: ethical or legal (n=5), physical (n=5), and psychological or psychiatric (n=2). No tools for bereavement or hospice care were identified. Studies have reported high acceptability of tools among users; however, few sources commented on usability among older adults.

**Conclusions:**

PCEOL patient portal tools are understudied. As medical care increasingly moves toward virtual platforms, future research should investigate the usability and acceptability of PCEOL patient portal resources and evaluate their impact on health outcomes.

## Introduction

Patient portals are secure websites that provide access to personal health information and health care services that often include web-based tools for medical visits, health records, and medications [1]. In addition to providing patients with access to their health records, health care organizations offer other digital health resources and functions through patient portals, including messaging with providers, general medical information, and prescription refills [2]. The growing adoption of portals shows that approximately 90% of health care organizations provide a portal system [3]. Through access to health records, patient portals promote self-management of health and disease and help improve patient-clinician communication [4-7].

Patients with chronic or serious illness may especially benefit from access to patient portals; in fact, having a chronic illness is a predictor of portal enrollment and use [8]. Older adults—80% of whom have at least one chronic illness [9]—are increasingly becoming the focus of chronic condition management and population health initiatives that involve the adoption and use of digital health technology, including patient portals. Although there are concerns about the low use of patient portals among this population [10-13], evidence also suggests a growing trend of internet and technology use among older adults [14,15]. This emphasizes the need to develop and tailor patient portals for the use of older adults. The rise of patient portal use also presents the opportunity to maximize patient empowerment, education, and patient-clinician communication among patients facing serious illnesses.

In light of the potential benefits of digital health technologies for older adults and individuals with chronic or serious illness, interest is also growing in digital health interventions targeted toward palliative care and end-of-life (PCEOL) patients, including the use of patient portals [16]. Palliative care focuses on pain and symptom management and improving quality of life for patients with serious illness and their families, and the Institute of Medicine has named access to these services as essential [17,18]. Despite the potential benefits of palliative care [19], issues with access to this specialized care persist because of barriers, including lack of availability and public awareness, particularly in rural areas [20,21]. Patient portals can be used to deliver advance care planning (ACP) education and provide access to materials for documenting advance directives, Medical Durable Power of Attorney (MDPOA), and health care proxies. Studies implementing ACP efforts through patient portals demonstrate patient acceptability as well as successful increases in ACP-related documentation [22-24]. Although this evidence is encouraging for the use of patient portals to promote PCEOL outcomes, there is also a need to understand the utility of patient portals for PCEOL patient education and resources and whether patient portal use is associated with other important PCEOL health outcomes, such as symptom management, hospice use (high quality palliative care near the end of life, typically in the last 6 months) [25], and documentation of care preferences.

As a first step in this line of research, we aim to conduct a scoping review of PCEOL patient portal research. The aims of this study are to (1) identify PCEOL patient portal tools available for patients and caregivers that are described and evaluated in the literature and (2) document patient and caregiver perspectives regarding these tools. By describing currently available tools and their respective user perspectives, this review intends to inform future research examining the association between tool use and PCEOL outcomes while guiding future PCEOL patient portal tool development.

## Methods

### Scoping Review

As little is known about the use of patient portals for the delivery of palliative and end-of-life care, a scoping review of the academic literature was conducted to identify sources describing currently available patient portal PCEOL tools. Our methods were aligned with methodological framework for scoping reviews by Arksey and O’Malley [26], which includes the following stages: identifying the research questions; identifying relevant studies; study selection; charting the data; and collating, summarizing, and reporting the results. The PRISMA (Preferred Reporting Items for Systematic Reviews and Meta-analyses) extension for Scoping Review checklist guided our reporting (Multimedia Appendix 1) [27]. Our scoping review goals and procedures were registered with the Open Science Framework on March 26, 2020 (DOI 10.17605/OSF.IO/N34JZ).

### Identifying Relevant Studies

To identify PCEOL patient portal tools described in the academic literature, we searched three databases (Ovid MEDLINE, CINAHL, and Web of Science) for peer-reviewed sources, including both qualitative and quantitative studies. The search terms included *patient portal* in combination with PCEOL terms such as *palliative care, hospice, end-of-life, terminal illness, cancer, advance directives, symptom management, ACP, grief*, and *caregiver support*. Our specific search strategy with exclusion terms is presented in Multimedia Appendix 2. The PCEOL search terms have previously been used to identify digital health solutions directed toward palliative care [28,29]. No time limits restricted the search, and only sources in English were included. We used Covidence, a web-based systematic review management program to remove duplicates, track citations, and screen references [30]. Our search was conducted in March 2020.

Sources were included in the scoping review if they (1) described a developed feature, resource, tool, or intervention that focused on at least one domain of PCEOL as defined by the National Coalition for Hospice and Palliative Care [31]; (2) targeted adults with serious illness (a health condition with a high risk of mortality impacting daily functioning or quality of life) [32] or their family and caregivers; and (3) were offered via a patient portal. The domains of palliative care include the following:

- Physical aspects of care including pain and symptom management, as well as quality of life assessment
- Psychological aspects of care, including care about anxiety, depression, stress, cognitive impairment, psychosocial support, coping skills, and support around patient or family grief and bereavement
- Social aspects of care, including patient and family education and caregiver support
- Spiritual and cultural aspects of care, including spiritual, existential, or religious support or attention to language, ritual, or dietary needs
- Care of the imminently dying, including end-of-life care and education
- Ethical and legal aspects of care, including ACP and documentation (appointment of MDPOA or health care proxy, and completion of advance directives)

To determine whether sources met the inclusion criteria, 2 coders (MPI and CV) independently reviewed the academic sources by title and abstract.

### Full-Text Review

Full texts of sources included by title and abstract were downloaded and further assessed for eligibility by 2 coders (MPI and CV). We determined whether the portal tool was targeted toward patients, caregivers, or both by reviewing the full text of the included sources for explicit mention of the intended users of the portal tool. The full text was also reviewed for a thorough description of the tool’s purpose and available features, apps, and content. Sources that presented a protocol or planned portal feature, did not provide a detailed description of the resources or content (eg, abstract and review articles), did not focus on a PCEOL domain, targeted a pediatric or non–seriously ill population, examined the user characteristics and perspectives of general portal use, were not published in English, described a tool that was not tethered to a patient portal (eg, free-standing website or mobile app), or for which the full text was not retrievable, were excluded from the review. Pediatric populations were excluded because of their different care needs, access to technology, and technological skills.

**Figure 1:**
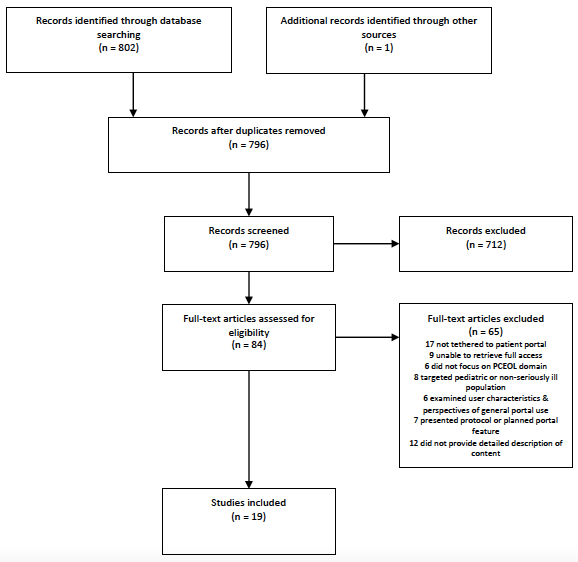
PRISMA (Preferred Reporting Items for Systematic Reviews and Meta-Analyses) flow diagram. PCEOL: palliative care and end-of-life.

The coders regularly reviewed the sources and discussed questions and possible disputes. Both coders agreed on the features included in the review, with a third reviewer (JDP) reconciling disputes. A summary of the search and screening processes is shown in Figure 1.

### Charting the Data

The full text of each of the included sources was reviewed, and the elements and features of the described PCEOL patient portal tool were abstracted by 2 reviewers (MPI and CV) into a Microsoft Excel spreadsheet. To ensure consistency, the reviewers double-extracted five articles before dividing the remaining articles. The abstracted data included the following components: portal name, health system user, developer (research team or organization), target illness and/or palliative care element, intended audience (patient, patient’s caregiver, or both), and a summary of the features. When described, patient and caregiver perspectives about the tool, including measurements and qualitative reports of acceptability or usability and user satisfaction, were abstracted.

### Collating, Summarizing, and Reporting the Results

We used an iterative and mixed inductive-deductive approach to summarize the charted information and results from the included papers to address our aims [26]. The charted information from each paper was reviewed extensively to identify similarities and differences in patient portal features, PCEOL domains, study population or targeted audience, and acceptability and usability findings (when available). The first, second, and senior authors (MPI, CV, and JDP) reviewed and agreed upon the thematic groupings of the results.

## Results

### Study Selection

The initial database search identified 796 unique sources that we screened by title and abstract (Figure 1). At this stage, we excluded 89.4% (712/796) of sources and reviewed 10.6% (84/796) of full-text sources. We excluded an additional 65 sources after full-text review because they presented a protocol or planned portal feature (7/65, 11%), examined user characteristics and perspectives of general portal use (6/65, 9%), described a tool that was not tethered to a patient portal (17/65, 26%), did not focus on a specific PCEOL domain (6/65, 9%), or was targeted at a pediatric or non–seriously ill population (8/65, 12%). At this stage, we also excluded sources that did not provide detailed descriptions of the tool’s intended audience, content, and/or features (12/65, 18%) and sources for which the full text was not retrieved through the 3 separate university libraries we had access to (9/65, 13%). Using these methods, we identified 18 sources eligible for inclusion. During the full-text review of a selected article [33], an additional article that met the inclusion criteria [34] was identified in the text, bringing the total number of included sources to 19.

### Identification of PCEOL Patient Portal Tools

We abstracted data from 19 sources published between the years 2013 and 2020 that describe 12 unique PCEOL patient portal tools (Table 1). The identified tools were developed by researchers and clinicians with the specific intent of enhancing PCEOL care. The identified PCEOL patient portal tools address the following domains: ethical and legal (n=5), physical (n=5), and psychological aspects of care (n=2). No tools address spiritual or bereavement support or hospice care education. Tools addressing the physical and psychological aspects of palliative care focus on quality of life and psychosocial support tools and are designed for cancer (n=6) and chronic kidney disease (n=1) patients. Patients are the intended audience for most PCEOL patient portal tools (n=9). Most tools were developed in an academic setting (n=11) and in the United States (n=9).

**Table 1:** Summary of included articles.

Study	Portal name	PCEOL^a^ domain	Sample size and characteristics	Study design	Developer	Summary of features	Summary of portal use	Promotion of use
Bajracharya et al [24]	PatientSite	Ethical or legal (ACP^b^)	200 patients; 63% women; 82% White; mean age 55 years (SD 15.16)	Evaluation of portal implementation	Beth Israel Deaconess Medical Center, Boston, MA, United States	A guided HCP^c^ interview delivered via patient portal that provides education on the HCP role, collects information necessary for the completion of an HCP form, allows patients to print the HCP form and submit their HCP form electronically. The HCP form may be incorporated into patient’s personal health record.	Of the participants that did not have an HCP listed before partaking in the interview, 78% submitted HCP information for clinician review. Of 200 patients, 139 submitted updated or new information about HCP overall.	N/A^d^
Tieu et al [35]	Mayo Clinic Patient Online Services	Ethical or legal (ACP)	2526 patients; 51% female; mean age 72 years (SD 5.8)	Randomized controlled intervention	Mayo Clinic, Rochester, MN, United States	Tool delivers a personalized, electronic message to patients with a link to ACP education and a state-specific advance directive form.	Only 5.5% of the intervention group and 2% of the control group completed and returned an AD^e^.	Patients randomized to intervention group received message encouraging them to complete AD; reminder was sent out 8 weeks after if patients had not completed.
Bose-Brill et al [34]	MyChart (operated through Epic)	Ethical or legal (ACP)	50 patients	Randomized controlled pilot intervention	The Ohio State University, Columbus, OH, United States	Tool delivers a secure message to patients consisting of an introduction to ACP and four ACP-related questions. The responses are automatically stored in the patient’s medical record and integrated into an ACP previsit planning algorithm focused on enhancing patient-provider communication surrounding ACP preferences.	In the intervention group, 10 of 23 participants completed ACP documentation compared with 1 of 25 participants in the control group.	N/A
Bose-Brill et al [33]	MyChart (operated through Epic)	Ethical or legal (ACP)	419 patients; 65% female; median age 61 years; range 50-93	Pragmatic trial	The Ohio State University, Columbus, OH, United States	Tool delivers a secure message to patients consisting of an introduction to ACP and four ACP-related questions. The responses are automatically stored in the patient’s medical record and integrated into an ACP previsit planning algorithm focused on enhancing patient-provider communication surrounding ACP preferences.	ACP documentation in EHR^f^ increased by 27% in intervention group, compared with 0.7% in control group. 78% of the intervention group read the initial message sent to them and 19% responded to at least one ACP-related question.	N/A
Lum et al [22]	MyHealth Connection (operated through Epic)	Ethical or legal (ACP)	2184 patients; 69% female; mean age 45 years; range 17-98	Quality improvement intervention	University of Colorado Hospital, Aurora, CO, United States	Tool provides patient-centered website for ACP education, secure messaging with an ACP support team, the ability to complete and sign an MDPOA^g^ form and to view the completed advance directive in the patient’s personal health record.	Over 15 months, 2814 patients used ACP tool; 89% completed MDPOA form, 2% called or sent web-based messages; 8% viewed MDPOA form without completing.	N/A
Jordan et al [23]	MyHealth Connection (operated through Epic)	Ethical or legal (ACP)	46 patients; 63% female; mean age 49 years	Exploratory qualitative study	University of Colorado Hospital, Aurora, CO, United States	Tool provides patient-centered website for ACP education, secure messaging with an ACP support team, the ability to complete and sign an MDPOA form and to view the completed advance directive in the patient’s personal health record.	Not collected	N/A
Brungardt et al [36]	MyHealth Connection (operated through Epic)	Ethical or legal (ACP)	105 older adult patients	Practice-based pilot initiative	University of Colorado Hospital, Aurora, CO, United States	Tool provides patient-centered website for ACP education, secure messaging with an ACP support team, the ability to complete and sign an MDPOA form and to view the completed advance directive in the patient’s personal health record.	At 1 year, 63 patients read ACP message and 17 had taken at least one ACP action step.	N/A
Portz et al [37]	My Health Manager (operated through Epic)	Ethical or legal (ACP)	24 older adult patients with multiple chronic conditions; 71% female; 79% White; mean age 78 years (SD 5.4)	Qualitative case study	Kaiser Permanente, Denver, CO, United States	Tool provides ACP education, ACP resources, and links to external website to complete advance directives.	Not collected	N/A
Dalal et al [38]	Patient-Centered Toolkit	Physical (QOL^h^)	119 admitted patients and 120 caregivers; 43% female; mean age 56 years	Evaluation of portal implementation	Brigham and Women’s Hospital, Boston, MA, United States	Tool allows patients with serious illness to navigate their plan of care during hospitalization. Patient can establish a single recovery goal and rate priorities, review medication and test results, securely message their care team, access educational content, and view discharge checklists, tailored safety tips, and reminders.	66% users inputted daily goal; 41% inputted overall goal (to be cured, to live longer, to be comfortable, other); 32% communicated care preferences; 64% provided real-time feedback for team. Goals, results, team members, medications, and messages were the most frequently visited pages. Problem education was the least frequently visited page.	N/A
Dalal et al [39]	Patient-Centered Toolkit	Physical (QOL)	55 patients preintervention, 40% female, 89% White; mean age 59 years (SD 12.8); 46 patients postintervention, 46% female, 83% White, mean age 58 years (SD 13.5)	Prospective pre- or postintervention study	Brigham and Women’s Hospital, Boston, MA, United States	Tool allows patients with serious illness to navigate their plan of care during hospitalization. Patient can establish a single recovery goal and rate priorities, review medication and test results, securely message their care team, access educational content, and view discharge checklists, tailored safety tips, and reminders.	Not collected	Participants were encouraged to enter recovery goals to the portal.
Kuijpers et al [40]	MyAVL or MijnAVL	Physical (QOL)	92 female patients with breast cancer; mean age 49 years (SD 11.4)	Pre- or posttest intervention	Netherlands Cancer Institute, Amsterdam, Netherlands	PRO^i^ collection and symptom management for patients with breast or non–small cell lung cancer. Patients can complete quality of life and physical activity questionnaires and receive personalized physical activity advice based on the questionnaire responses.	Overview of appointments and EMR^j^ were accessed most frequently; mean number of log-ins for on-treatment is 10.9 with mean of 11.3 minutes. Mean number of log-ins for off-treatment is 5.6 with mean of 15.2 minutes.	N/A
Groen et al [41]	MyAVL (MijnAVL)	Physical (QOL)	37 patients with lung cancer; 47% women; 100% White; mean age 59 years (SD 8.4); range 40-76 years	Feasibility intervention	Netherlands Cancer Institute, Amsterdam, Netherlands	PRO collection and symptom management for patients with breast or non–small cell lung cancer. Patients can complete quality of life and physical activity questionnaires and receive personalized physical activity advice based on the questionnaire responses.	Mean number of log-ins over 4-month study period—11.2 with mean duration of 12.9 minutes. Overview of appointments, access to EMR, and questionnaires were the most accessed.	N/A
Wagner et al [42]	MyChart (operated through Epic)	Physical (QOL)	636 female patients with cancer; 78% White; age mean 55 years (SD 12.8); range 21-90 years	Clinical quality improvement initiative	PROMIS^k^, Northwestern University (Chicago, IL) and US Department of Health and Human Services (Washington, DC), United States	PRO collection and symptom management for patients with cancer. Delivers electronic PRO assessments and other assessments to identify psychosocial concerns, informational and nutritional needs. The results are immediately populated to the patient’s EHR. This integration allows automated triage to multidisciplinary care team.	80% of patients read the initial MyChart message asking for e-PRO^l^ assessment completion; about 33% completed the entire assessment. 90% of the patients that completed the assessment did so at home.	e-PRO assessments were sent before scheduled outpatient appointments through MyChart. Those who did not compete the assessment at home were provided with an iPad to complete the assessment during appointment check-in.
Garcia et al [43]	MyChart (operated through Epic)	Physical (QOL)	3521 patients with cancer; 68% female; 76% White; mean age 57 years (SD 13.39)	Clinical quality improvement initiative	PROMIS, Northwestern University (Chicago, IL) and US Department of Health and Human Services (Washington, DC), United States	PRO collection and symptom management for patients with cancer. Delivers electronic PRO assessments and other assessments to identify psychosocial concerns, informational and nutritional needs. The results are immediately populated to the patient’s EHR. This integration allows automated triage to multidisciplinary care team.	Not collected	e-PRO assessments were sent before scheduled outpatient appointments through MyChart. Those who did not compete the assessment at home were provided with an iPad to complete the assessment during appointment check-in.
Brant et al [44]	Carevive Care Planning System	Physical (QOL)	121 female patients with gynecologic or breast cancer; 83% White; age mean 56 years (SD 10.94); range 28-81	Mixed methods pilot study	Carevive, United States	PRO collection and symptoms management for patients with breast or gynecologic cancer. Collects PRO and integrates responses and clinical data to create a tailored care plan that provides clinical decision support and self-management advice to patient and caregivers.	Not collected	N/A
Hazara et al [45]	Renal PatientView	Physical (QOL)	190 patients with chronic kidney disease; 34% female; median age 53 years; range 19-86	Quality improvement initiative	Renal Patient Exchange Group, United Kingdom	PRO collection and symptom management for patients with chronic kidney disease. Patients can document and monitor symptoms and other health indicators and access educational resources.	Between 2009-2013, tool had 421 registered users and 54% were active.	N/A
Hudson et al [46]	Renal PatientView	Physical (QOL)	10 patients with chronic kidney disease; 20% female; age mean 59 years	Ethnographic qualitative study	Renal Patient Exchange Group, United Kingdom	PRO collection and symptom management for patients with chronic kidney disease. Patients can document and monitor symptoms and other health indicators and access educational resources.	Not collected	N/A
Pai et al [47]	Provider	Psychological or psychiatric	22 male patients with prostate cancer; 95% White; mean age 64 years (51-76)	Nonexperimental posttest only	British Columbia Cancer Agency and the School of Health Information Science at the University of Victoria, Victoria, BC, Canada	Psychosocial support for patients with prostate cancer. Allows patients to view a summary of prostate cancer diagnosis, cancer treatment received, and monitor health indicators. Patients can also access a treatment decision support tool, a questionnaire on distress level, education on prostate cancer and its treatments, and a tailored clinical trial and research screening tool.	Mean registered log-ins over 6 months was 3.4. Medical records, appointment viewer, and PSA^m^ monitoring tool were accessed most frequently by users.	N/A
Nahm et al [48]	CaS-PET^n^	Psychological or psychiatric	30 patients with cancer; 77% female; 33% White; mean age 56 years (SD 13.6)	One group pre- or posttest pilot study	University of Maryland Medical Center, Baltimore, MD, United States	Psychosocial support for patients with cancer. Provides 12-week content with six modules including transition to survivorship, nutrition, exercise, cancer and relationships, fear and mental health, and stress management using mindfulness. There are goal setting activities, discussion boards, and virtual libraries at the end of each module.	Not collected	Biweekly follow-up messages were sent out after initial message.

^a^PCEOL: palliative care and end-of-life.

^b^ACP: advance care planning.

^c^HCP: health care proxy.

^d^N/A: not applicable.

^e^AD: advance directive.

^f^EHR: electronic health record.

^g^MDPOA: Medical Durable Power of Attorney.

^h^QOL: quality of life.

^i^PRO: patient-reported outcome.

^j^EMR: electronic medical record.

^k^PROMIS: Patient-Reported Outcomes Measurement Information System.

^l^e-PRO: electronic patient-reported outcome.

^m^PSA: prostate-specific antigen

^n^CaS-PET: Cancer Survivorship Patient Engagement Toolkit.

### Features of PCEOL Patient Portal Tools

#### Ethical or Legal

All five tools addressing ethical and legal aspects of care center on ACP, including ACP education and the opportunity to complete documentation of advance directive, or of an appointed MDPOA or health care proxy. The ACP tools in each of these patient portals provide education and a means to facilitate documentation of advance care preferences. We identified two tools (PatientSite and MyChart) that use a guided, patient-centered questionnaire to enable the completion of an advance directive [24,33,34]. Although most of the tools allow users to complete and submit an advance directive that directly integrates into the electronic health record [22,23,35,36], we identified one tool based on My Health Manager (a Kaiser Permanente portal built through Epic software) that links users to an external website to complete the advance directive [37].

#### Physical

The identified PCEOL patient portal tools addressing physical aspects of care focus on improving the quality of life of patients with serious illness. Four of the five tools identified allow users to complete patient-reported outcome assessments and other questionnaires electronically [42-46]. The results of the assessments were automatically integrated into the electronic medical record and then used to generate an action. One of the tools incorporated the Patient-Reported Outcomes Measurement Information System (PROMIS), which employs computer-adaptive tests to measure patient-reported symptoms and health-related quality of life across various medical conditions [42,43]. Wagner et al [42] administered an assessment that included a subset of PROMIS computer-adapted tests measuring pain interference, fatigue, physical function, depression, and anxiety and a checklist to identify psychosocial, informational, and nutritional needs [43] in patients with gynecological cancers. For each outcome, a score above a specific threshold triggers an alert to the appropriate member of the multidisciplinary care team for further evaluation. This assessment was also conducted in a larger cohort of adult medical oncology outpatients [42].

Similarly, MyAVL (MijnAVL), a patient portal for patients with breast and non–small cell lung cancer in the Netherlands, allows users to complete quality of life and physical activity questionnaires. Users are provided with tailored physical activity advice based on their responses. The commercially developed Carevive Care Planning System allows for the collection of patient-reported outcomes in patients with breast and gynecological cancers [44]. Targeted at both patients and caregivers, the users’ responses were used to create a care plan that included clinical decision support and patient education and self-management resources. Renal PatientView, developed in the United Kingdom, was the only tool identified in patients with chronic kidney disease [45,46]. Renal PatientView allows users to monitor symptoms and other health indicators (eg, weight, blood pressure, and blood glucose) electronically and access educational content specific to their diagnosis.

Only one of the tools addressing quality of life, the Patient-Centered Tool Kit (PCTK), was designed for use during acute hospitalization [38,39]. The PCTK has been studied in patients admitted to medical intensive care and oncology units and their caregivers. Integrated with the patient’s electronic health record, the tool allows users to establish a single recovery goal that can be updated throughout hospitalization. Users can also access educational content, predischarge checklists, and navigate their plan of care.

#### Psychological

Two of the PCEOL patient portal tools addressed the psychological aspects of care, primarily providing resources for psychosocial support. The British Columbia Cancer Agency developed the *Provider* tool for patients with prostate cancer. This tool allows users to complete a questionnaire on distress levels and access treatment decision support resources [47]. The tool also provides users with education on prostate cancer, its treatment, and a personalized clinical trial screening tool. The Cancer Survivorship Patient Toolkit (CaS-PET) was developed to provide survivorship care plans for patients with cancer [48]. This tool uses a biweekly follow-up contact via secure messaging. Each message asks about the patient’s general condition and support needs and includes links to well beyond cancer, a web-based resource with six modules that include transition to survivorship, nutrition, exercise, cancer and relationships, fear and mental health, and stress management using mindfulness. The modules incorporate goal setting, discussion boards, and virtual libraries.

### Information on Use or Promotion of Use

Portal use measures were described for nearly all (n=10) patient portal tools in at least one study. Table 1 illustrates these findings in detail. Portal use was most often measured by the mean number of log-ins and duration in minutes [40,41,47], number of registered users [45], features accessed [38,40,41,47], or completed activities such as completing an AD or other ACP-related documentation [22,24,33-36] or inputting a daily goal [38].

Overall, an overview of appointments and medical records were the most frequently accessed features for portals [38,40,41,47]. The Patient-Centered Toolkit assessed that education on patients’ *problem* or condition was the least visited feature; however, this could be explained by the study’s in-patient setting where the patients and caregivers had more frequent access to providers [38,39].

Studies for four of the portals specifically described methods of promoting portal use, either by reminders via electronic messages [35,42,48] or in-person [39]. The impact of these promotions of use is mixed; two of the studies did not collect data on portal use to correspond to their promotion efforts. After sending electronic messages prompting patients to complete their e-PRO assessment before their scheduled appointment, Wagner et al [42] found that 80% of recipients read the message, while 33% of them completed the electronic patient-reported outcomes (e-PRO) assessment.

### User Perspectives

Usability, acceptability, and user satisfaction were formally evaluated for nine of the identified PCEOL patient portal tools (Table 2). Only two studies have specifically assessed the user perspectives of older adults [37,49]. In general, usability and acceptability rankings were high, and users were satisfied with the tools. Users valued tools that allowed the electronic collection of patient-reported outcomes and symptoms. Aside from their perceived convenience, these tools allow users to gain a sense of control over their health, improve symptom management, and access novel resources [40,41,44,47,48].

Users viewed these tools as a means of improving communication. In particular, the integration of tools with electronic medical records was valued. In ACP tools, users appreciated that the advance directive forms were easily retrievable and viewable for their providers and family [23,24,37]. The tools addressing quality of life and psychosocial support were also perceived to improve communication with the care team [39,44,47,48]. There was evidence of increased care plan concordance between patients [39], and users noted that the tools better prepared them for their clinical visits [44,47,48]. Users of the CaS-PET felt that the tool helped them determine what questions to ask during their medical appointments.

**Table 2:** Portal tool usability and satisfaction.

Portal tool	Usability sample size (overall sample size)	Data collection	Mean usability or satisfaction scores or qualitative comments	Comments for improvement
PatientSite [24]	74 (200)	Qualitative feedback	Patient participants had positive feedback on usability, tool prompted patients to take the time to think about their HCP^a^, gave them an opportunity to improve their HCP information, and helped patients tackle this difficult topic	Patients made minor suggestions to improve the capability to edit form directly, to improve the ease of printing the form, and to add ability to appoint an additional HCP.
MyHealth Connection (Epic) [22,23]	11 (2814) 46 (46); 63% female; mean age 49 years	SUS^b^ Qualitative feedback	89 (a total of 70 or greater is typically considered acceptable for usability) Patients were generally satisfied with the ease of use and were likely to recommend using the tool for ACP^c^ documentation to others.	N/A^d^
My Health Manager (Epic) [37]	24 (24); 71% female; 79% White; mean age 78 years (SD 5.4)	Qualitative feedback (focus groups)	Most participants reported interest in having AD^e^ documentation features available in the electronic medical record	N/A
PCTK^f^ [38]	18 (239); 10 patients; 50% female; 80% White; 70% >51 years 8 caregivers; 75% female; 87% White; 87% >51 years	SUS	74 (a total of 70 or greater is typically considered acceptable for usability)	Feedback included suggestion for improving technical features and displays to enhance clinical communication.
MyAVL (MijnAVL) [40,41]	28 (37) 92 (92); all female; mean age 49 years (SD 11.4)	WUS^g^; UTAUT^h^ WUS; UTAUT	3.9 (maximum score of 5, indicated the highest level of satisfaction); 93% reported tool easy to use, 69% reported tool valuable addition to health care experience 3.8; 75% reported tool easy to use	N/A Focus groups expressed overall satisfaction with portal features, however expressed desire for educational content to be more tailored to their specific condition
Carevive Care Planning System [44]	94 (121)	SUS	83 (a total of 70 or greater is typically considered acceptable for usability)	N/A
Renal PatientView [45,46]	190 (190) [45]; 34% female; median age 53 years; range 19-86 years 10 (10); 20% female; mean age 59 years	Investigator-developed questionnaire Qualitative feedback	45% of inactive users cited computer or password issues as primary issue for nonuse; 37% of inactive users said portal did not anything to their relationship with clinicians Patients found the portal valuable to help prepare themselves and family for changes in care and had better understanding of how their symptoms, blood results, and physiological changes were connected; able to better involve family in care	N/A N/A
Provider [47]	22 (22); all male; 95% White; mean age 64 years; range 51-76 years	Questionnaire not specified	88% of respondents rated overall satisfaction as excellent or very good; 88% of respondents would continue to use the tool	N/A
CaS-PET^i^ [48]	30 (30); 77% female; 33% White; mean age 56 years (SD 13.6)	Health Website Usability Questionnaire subscale; open-ended questions	Most patients (n=22) found the portal helpful and reported having helpful information or that it helped them stay healthy	N/A

^a^HCP: health care proxy.

^b^SUS: Systematic Usability Scale.

^c^ACP: advance care planning.

^d^N/A: not applicable.

^e^AD: advance directive.

^f^PCTK: Patient-Centered Tool Kit.

^g^WUSQ: Website User Satisfaction.

^h^UTAUT: Unified Theory of Acceptance and Use of Technology.

^i^CaS-PET: Cancer Survivorship Patient Engagement Toolkit.

## Discussion

### Principal Findings

Patient portals may offer accessible avenues for keeping individuals with serious illness and their caregivers connected to PCEOL resources. Although the use of patient portals among patients with serious life-limiting illness is of growing interest, previous studies suggest that patients with serious illness and their caregivers are potentially extensive users of patient portal tools that are commonly available, particularly to access care team information, review laboratory results, and obtain medical records [50,51]. Interest in using patient portals among this population may be high and potentially an excellent venue for providing PCEOL-specific information and resources. This scoping review of 19 articles described available PCEOL features via patient portals tethered to electronic health records. Overall, the review highlighted some of the ways patient portals are offering PCEOL resources, as well as opportunities for future work to leverage patient portals to increase access and awareness of PCEOL care.

The results identified 12 unique patient portal resources supporting the following PCEOL domains: ethical or legal (in the case of this review, exclusively ACP education; n=5), physical (including symptom monitoring, goals of care, and patient-reported outcomes; n=5), and psychological support (n=2). Most resources were targeted toward patient audiences (n=9), with the remainder being targeted toward caregivers, or both patients and caregivers. Only two resources focused specifically on the user characteristics of older adults [37,49], which indicates a clear opportunity to improve our understanding of use and usability in this high-need patient population.

Evidence demonstrates that patients living with serious illnesses and their family members and caregivers can benefit from palliative care [52]. However, there are nearly 1 million patients who are admitted to the hospital annually who could benefit from palliative care and yet do not receive this specialized care [53]. In addition, people living in rural communities have less access to palliative care services than their urban counterparts [54]. Although we recognize that palliative care is highly personalized, patient portals offer the opportunity to leverage technology to promote access and education regarding PCEOL issues. Incorporating PCEOL resources into patient portal features may serve to introduce or supplement specialty palliative care services or bridge the gap where patients have no access to palliative care.

Furthermore, the role of digital health solutions, including patient portal resources, is more important than ever in the wake of the COVID-19 pandemic. Health systems across the United States, and the globe, are forced to incorporate digital care as part of their regular repertoire of available patient care as safety precautions and social distancing have become the norm [55,56]. Individuals who are appropriate for palliative care are highly vulnerable and must be especially considered when addressing how to bring quality virtual health care to individuals during a pandemic. These patients have an increased risk of contracting the virus and experiencing related complications (eg, hospitalization or death) because of their age and health status. They have also been uniquely impacted by the pandemic in that they still require continuous disease management and may experience disproportionate exacerbations of psychosocial needs [57]. Finally, people are dying or experiencing bereavement in isolation at higher rates in the wake of the pandemic [58,59], emphasizing the need for creative palliative care solutions, including better access to PCEOL information and resources for individuals with serious illness and their loved ones.

Nearly all the portals included in this review focused on quality of life, symptom management, or ACP education or completion of advance directives. ACP may be better leveraged through patient portals than other resource types given its more limited and specific goal than some of the more complex and nuanced needs and components inherent in PCEOL care. Similarly, PROMIS tools and other electronic mechanisms for patient-reported outcomes are well suited for patient portals.

Although the legal and physical aspects are essential pieces of quality and holistic PCEOL services, equally important are the psychological and spiritual aspects, as well as end-of-life education around hospice. Only two of the portals reviewed focused on the psychological needs of patients with serious illness [47,49]. None of the portals included in the review offered features addressing grief and bereavement or information about hospice or comfort care. Despite the evidence supporting the benefits of hospice care [60-62], lack of information and understanding remains one of the most common barriers to hospice use [63-65]. Furthermore, bereaved individuals and families often benefit from educational resources related to the grief process [66]. Future research and development on portal tools for patients with serious illness and their family members should emphasize psychological and spiritual PCEOL domains and consider tools and educational resources regarding grief, bereavement, and hospice.

It is important to emphasize the special consideration of older adult patients with serious illness and patient portal tool development, availability, and adoption among PCEOL populations. In total, 80% of older adults in the United States have one or more chronic conditions, many of which can lead to serious illness [9]. As the older adult population continues to grow, and subsequently the number of older adults experiencing serious illness increases, the focus of patient portals’ development and research must continue to include the perspectives and needs of patients aged ≥65 years. Despite the currently low adoption rate of portals by older patients and caregivers, research demonstrates that there is an interest in such tools among this group [37,67], an interest that might well be addressed by improving the design and availability of solutions tailored to and especially suited to the needs of older patients [68]. Future research on PCEOL patient portal development, use, and acceptability should explicitly incorporate older adult needs and feedback.

### Limitations

To the best of our knowledge, this scoping review is the first to identify and describe patient portals that offer PCEOL features. However, this is not without limitations. First, the review only included results from academic literature and may be missing commercially available portals that were developed by industry without concurrent publication in the peer-reviewed literature. In addition, we did not evaluate the full text of articles in which we were unable to retrieve access, potentially overlooking additional PCEOL patient portal resources. Although some of the articles described the efficacy on clinically meaningful outcomes, we did not address this information as it was beyond the scope of our scoping view. Future research should focus on the efficacy of such tools. Finally, this review had a small sample size of articles (n=19) covering 12 different patient portals that are predominantly US based, potentially limiting the results to a Westernized PCEOL perspective.

### Conclusions

The scoping review highlighted an important gap in PCEOL resources offered via patient portals linked to electronic health records, as well as opportunities for leveraging patient portals as a means of offering education and support for patients with serious illness. The results suggest that there are ongoing efforts to offer various PCEOL supports through patient portals, particularly with a focus on ACP. However, our review resulted in a small number of portals that met our criteria, of which included only a few of the expansive elements of PCEOL care. Future research and development of patient portals would benefit from offering comprehensive PCEOL features to increase access and education for patients experiencing serious illness.

## Appendix

Multimedia Appendix 1 Preferred Reporting Items for Systematic Reviews and Meta-Analyses extension for Scoping Review (PRISMA-ScR) checklist.

Multimedia Appendix 2 Scoping review search terms.

## Abbreviations

ACP: advance care planning
MDPOA: Medical Durable Power of Attorney
PCEOL: palliative care and end-of-life
PRISMA: Preferred Reporting Items for Systematic Reviews and Meta-Analyses
PROMIS: Patient-Reported Outcomes Measurement Information System
