# Supplementary material for: Patient Portals to Support Palliative and End-of-Life Care: Scoping Review
Source: J Med Internet Res. 2021 Sep 16;23(9):e28797. doi: 10.2196/28797 (PMC8485198; doi:10.2196/28797)
Supplement: Multimedia Appendix 2 [file jmir_v23i9e28797_app2.pdf]

Appendix 2: Scoping Review Search Terms

Patient portal terms:
1. exp "Electronic Health Records"/ and portal*
2. exp "Health Records, Personal"/ and portal*
3. exp "Patient Portals"/
4. (patient or health or electronic or internet or web*) adj2 portal*
5. ((electronic or personal) adj2 (health or medical) adj2 record*) and portal*
6. (EHR or EMR or EPHR or PHR) and portal*

Palliative, hospice, end-of-life care, and other specified terms:
1. exp "Advance Care Planning"/
2. exp "Hospice and Palliative Care Nursing"/
3. exp "Hospice Care"/
4. exp "Hospices"/
5. exp "Palliative Care"/
6. exp "Palliative Medicine"/
7. "Terminal Care"/
8. exp "Terminally Ill"/
9. exp "Grief"/
10. exp "Chronic Disease"/
11. exp "Neoplasms"/
12. advance? adj2 (care or healthcare or medical) adj1 plan*
13. advance? directive*
14. (health* or medical) adj2 power adj2 attorney
15. living will*
16. end of life
17. hospice*
18. palliat*
19. terminal* adj1 (care or ill*)
20. (advanced or chronic* or serious) adj2 (illness* or condition* or disease*)
21. cancer*
22. caregiver support
23. grief
24. oncolog*
25. symptom management

Excluded terms:
1. portal vein
2. portal venous
3. portal hypertension
4. portal imaging
